# Remoteness and emergency care pathways for ambulance-attended atrial fibrillation: a population-based linked cohort study

**DOI:** 10.1016/j.ijcha.2026.101997

**Published:** 2026-08-20

**Authors:** Malanka Lankaputhra, Emily Mahony, Luke P. Dawson, Aleksandr Voskoboinik, Riley Batchelor, Vishal Goel, Mark Horrigan, Peter Kistler, Jonathan Kalman, Tegwyn McManamny, David Anderson, Emily Nehme, Ziad Nehme, Dion Stub, Jocasta Ball

**Affiliations:** a School of Public Health and Preventive Medicine, Monash University, Melbourne, Victoria, Australia; b Department of Cardiology, The Alfred Hospital, Melbourne, Victoria, Australia; c Ambulance Victoria, Melbourne, Victoria, Australia; d Baker Heart and Diabetes Institute, Melbourne, Victoria, Australia; e Duke University Health, Durham, NC, USA; f St Vincent's Clinic, Melbourne, Victoria, Australia; g Austin Hospital, Melbourne, Victoria, Australia; h Department of Cardiology, The Royal Melbourne Hospital, Melbourne, Victoria, Australia; i Department of Medicine, University of Melbourne and Baker Research Institute, Melbourne, Victoria, Australia; j Department of Paramedicine, Monash University, Melbourne, Victoria, Australia; k Monash Alfred Baker Centre for Cardiovascular Research, Melbourne, Victoria, Australia; l Intensive Care Services, The Alfred Hospital, Melbourne, Victoria, Australia

**Keywords:** Atrial fibrillation, Emergency medical services, Rural health, Remoteness, Prehospital care, Linked data

## Abstract

**Background:**

Atrial fibrillation (AF) frequently requires emergency medical services (EMS), but acute-care pathways may differ by remoteness. We examined ambulance-attended AF care and outcomes across Victoria, Australia.

**Methods:**

We conducted a population-based linked cohort study of first ambulance-attended AF events from Jan 1, 2015, to June 30, 2019. Residence was classified using ARIA+ as major cities, inner regional, or outer regional/remote. We estimated incidence rate ratios (IRRs) and used adjusted logistic regression to compare care pathways, rhythm-control procedures, EMS re-attendance, and mortality.

**Results:**

Among 16,372 events, 11,914 (72.8%) occurred in major cities, 3691 (22.5%) in inner regional areas, and 767 (4.7%) in outer regional/remote areas. Annualised incidence was higher in inner regional (IRR 1.31, 95% CI 1.27–1.36) and outer regional/remote areas (IRR 1.25, 95% CI 1.16–1.35) than in major cities. Transport to hospital exceeded 99% across groups. Transport longer than 60 min increased with remoteness (0.7%, 6.4%, and 11.2%), whereas offload delay was concentrated in major cities (69.6%, 46.2%, and 35.9%). Rhythm-control procedures, EMS re-attendance for AF, and mortality were similar after adjustment, whereas socioeconomic disadvantage was associated with fewer rhythm-control procedures and higher mortality independently of remoteness.

**Conclusions:**

Ambulance-attended AF is more frequent outside major cities and imposes greater transport burden, while metropolitan care is more affected by offload delay. Similar outcomes should not be interpreted as similar care. AF pathways should target ED flow in cities and prehospital triage and follow-up outside cities.

**Research in context:**

Evidence before this study

AF is a major contributor to emergency care use, hospitalisation, stroke, and mortality. Prior statewide Victorian studies have described the overall ambulance and ED burden of AF, while international literature suggests that rural patients experience barriers to AF diagnosis, specialist care, and longitudinal management. However, few population-based linked data studies have compared the full prehospital-to-hospital pathway for ambulance-attended AF by remoteness of residence.

Added value of this study

This study links statewide EMS, ED, hospital, and mortality data to show that ambulance-attended AF incidence is higher in regional Victoria and that prolonged transport is substantially more common outside major cities. Conversely, offload delay is concentrated in major cities. Despite these pathway differences, adjusted EMS re-attendance, mortality, and rhythm-control procedure rates were similar across remoteness categories, whereas area-level socioeconomic disadvantage was associated with fewer rhythm-control procedures and higher mortality regardless of where patients lived.

Implications of all the available evidence

Statewide AF pathways should not assume that the same bottleneck applies everywhere. Metropolitan strategies should address ED congestion and offload delay, while rural implementation should prioritise transport burden, prehospital risk stratification, and reliable follow-up after ED discharge or non-transport. Socioeconomic disadvantage cuts across both settings and warrants attention in its own right.

## 1 Introduction

Atrial fibrillation (AF) is the most common sustained cardiac arrhythmia and is independently associated with stroke, heart failure, hospitalisation, reduced quality of life, and mortality [1], [2], [3]. Its population burden is increasing as communities age and as detection improves through greater access to electrocardiography, wearable devices, and longitudinal monitoring. AF care also extends beyond the acute episode. Effective management requires stroke-risk assessment, anticoagulation when indicated, symptom control, decisions about rate or rhythm control, and timely follow-up after destabilising presentations [1], [2], [3].

Emergency medical services (EMS) and emergency departments (EDs) manage a substantial part of this burden. A previous statewide Victorian cohort identified 16,525 first ambulance-attended AF events among 2,613,056 EMS attendances between January 2015 and June 2019, about 0.6% of the entire EMS caseload; about 99% of these patients were transported to hospital and almost half received no prehospital treatment [4]. Population-based ED data also demonstrate a growing AF presentation burden [5]. In the present cohort, most transported patients were either discharged directly home from the ED or managed in an ED short-stay unit rather than admitted to a ward. Near-universal transport, frequent ED discharge or short-stay management, and the need for ongoing follow-up together make AF a useful tracer condition for examining whether acute cardiovascular pathways are matched to local system constraints.

Remoteness is likely to affect both the need for EMS and the experience of care. Rural and regional communities may have older age structures, higher cardiovascular risk, reduced access to specialist electrophysiology and cardiology services, fewer ambulatory diagnostic options, and longer distances to hospitals capable of acute assessment or rhythm-control procedures [6], [7]. For patients, a seemingly simple ED assessment can require prolonged ambulance transport, separation from local supports, and a return journey after discharge. For EMS systems, long-distance transport removes ambulance capacity from the originating community. Metropolitan areas face a different pressure, because high ED demand and ambulance offload delay can reduce fleet availability even when travel distances are shorter [8].

These issues are particularly relevant for health systems that must deliver standardised acute care across both dense urban centres and sparsely populated regions. A single statewide protocol may improve consistency, but it may also miss local barriers. A pathway designed around metropolitan access block may not address rural travel burden, while a pathway designed to reduce rural transport may not solve urban offload delay. Understanding where the pathway differs is therefore a prerequisite for targeted redesign.

Despite these differences, few population-based linked data studies have compared the complete prehospital-to-hospital pathway for ambulance-attended AF by remoteness of residence. We therefore used statewide linked EMS, ED, hospital admission, procedure, and mortality data to estimate incidence and compare prehospital intervals, hospital pathways, rhythm-control procedures, EMS re-attendance for AF, and mortality across remoteness categories in Victoria.

## 2 Methods

### 2.1 Study design and data sources

We conducted a retrospective, population-based linked cohort study in Victoria, Australia. At the end of each case, Ambulance Victoria paramedics complete an electronic patient care record containing the on-scene clinical assessment, physiological measurements, treatments, and case time stamps. These records were linked to the Victorian Emergency Minimum Dataset (VEMD), the Victorian Admitted Episodes Dataset (VAED), and the Victorian Death Index. The VEMD captures administrative and clinical information for public hospital ED presentations, including ED arrival and departure times, diagnoses, and disposition, and therefore defines the ED segment of the pathway. The VAED captures demographic, diagnostic, procedural, and administrative information for admitted episodes in public and private hospitals, and defines the inpatient segment, including the rhythm-control procedures reported here. The Victorian Death Index records registered deaths. Ambulance Victoria is the single statewide EMS provider for about 6.5 million residents across metropolitan, regional, and remote areas.

The cohort comprised consecutive adults aged 18 years or older attended by EMS for AF between Jan 1, 2015, and June 30, 2019. AF was identified from the paramedic on-scene assessment and electronic patient care record when paramedics documented either a primary or secondary diagnosis of “atrial fibrillation”, or a primary diagnosis of “arrhythmia” with an ECG-confirmed initial rhythm of AF. The cohort was therefore defined prospectively from prehospital clinical documentation rather than retrospectively from the hospital discharge diagnosis or dispatch code. AF was the initial paramedic-recorded rhythm in 93.0% of included events, and a final primary assessment of arrhythmia was recorded in 73.5%. Among transported events with a linked ED record, the principal ED diagnosis was AF or atrial flutter in 53.4% and another arrhythmia in 8.6%, with the remainder coded to a precipitating or coexisting condition such as heart failure, chest pain, or pneumonia. Trauma cases, inter-hospital transfers, non-emergency ambulance attendances, and patients younger than 18 years were excluded; patients with pre-existing AF were not excluded. Only the first AF-related EMS presentation per linked individual was retained as the index event, with subsequent AF-related EMS contacts counted only as re-attendance outcomes. No additional exclusion criteria were applied. Records without valid remoteness data were excluded only from remoteness-stratified analyses.

### 2.2 Remoteness classification

Remoteness of usual residence was classified using ARIA+, as operationalised through the Australian Statistical Geography Standard Remoteness Areas (Edition 3) [9]. ARIA+ measures road-network access from populated localities to service centres; lower scores indicate greater access. Five ASGS categories were initially available: Major Cities, Inner Regional, Outer Regional, Remote, and Very Remote.

For analysis, categories were collapsed into three groups: major cities, inner regional areas, and outer regional/remote areas. Outer Regional, Remote, and Very Remote were combined because the latter categories contain small numbers in Victoria and because the clinical question was whether increasingly non-metropolitan residence is associated with different EMS pathways and outcomes. Remoteness was assigned from usual residential address, meaning that it reflects patients' place of residence rather than the exact location of symptom onset.

### 2.3 Socioeconomic status

Socioeconomic status was operationalised as an area-level measure using the Index of Relative Socio-economic Disadvantage (IRSD), one of the Australian Bureau of Statistics Socio-Economic Indexes for Areas (SEIFA) derived from the 2016 Census. IRSD was assigned from residential postcode and summarises relative disadvantage using Census characteristics including income, employment, and educational attainment. Published IRSD deciles were collapsed into quintiles for analysis, with quintile 1 denoting the greatest relative disadvantage and quintile 5 the least. IRSD was unavailable for 1385 events (8.5%), which were excluded from adjusted models. IRSD describes the socioeconomic characteristics of an area and should not be interpreted as an individual-level measure [10].

### 2.4 Outcomes

Process measures included response time, scene duration, transport duration, total prehospital time from emergency call to ED arrival, ambulance time at ED, and ED length of stay. Offload delay was defined as ambulance time at ED of 40 min or longer, consistent with established transfer benchmarks and prior ambulance offload research [8], [11]. Hospital admission was identified from admitted-care data and included short-stay unit care where captured. ED discharge without hospital admission was reported among transported events with a linked public ED record.

Clinical outcomes included EMS re-attendance for AF within 30 days and 6 months, death within 30 days and 12 months, and rhythm-control interventions. Direct-current cardioversion and catheter ablation were identified from hospital admission procedure codes at any point between the index EMS-attended event and the end of available follow-up, including procedures performed during the index episode. Because follow-up ended on a fixed date, its length varied by index date (median 2.7 years, IQR 1.6 to 3.9 years), and rhythm-control procedures were therefore also examined within a fixed 12-month window as a sensitivity analysis. EMS re-attendance was restricted to AF presentations and should not be interpreted as all-cause health-care use.

### 2.5 Incidence and statistical analysis

Population-based incidence was calculated as events per 100,000 person-years using Australian Bureau of Statistics estimated resident population denominators stratified by remoteness category; person-time for 2019 was adjusted for the half-year observation window [12]. Incidence rate ratios (IRRs) were estimated with Poisson regression models including population offsets and adjustment for calendar year.

Baseline characteristics and process measures were summarised by remoteness category. Continuous variables are presented as median [IQR] or mean (SD), as appropriate; categorical variables are presented as number and percentage. Numbers use the decimal point throughout, the comma is reserved for thousands separators, and interquartile ranges and confidence intervals are separated by a dash. High thromboembolic risk was defined as a CHA2DS2-VASc score of at least 2 in men and at least 3 in women. Group comparisons used Kruskal-Wallis tests or chi-square tests.

Associations between remoteness and binary outcomes were estimated using multivariable logistic regression and reported as adjusted odds ratios (aORs) with 95% CIs and *P* values. In response to concerns about overlapping composite indices, the final models did not include CHA2DS2-VASc category or Charlson Comorbidity Index. Models instead adjusted for age, sex, pre-existing AF, coronary artery disease, chronic obstructive pulmonary disease, hypertension, heart failure, diabetes, cerebrovascular disease, peripheral vascular disease, IRSD quintile, and calendar year. Three secondary analyses were prespecified for this revision. First, the exposure was reversed and associations between IRSD quintile and each outcome were estimated using the same covariates. Second, remoteness estimates were compared between models with and without IRSD to assess how much of any geographic difference was attributable to socioeconomic composition. Third, hospital admission and rhythm-control models were repeated with standard errors clustered by receiving ED campus, to allow for correlation between events managed at the same destination hospital. No imputation was performed; adjusted analyses used complete cases, and model sample sizes are reported in the Supplementary Appendix. Analyses were conducted in R/RStudio and Python (statsmodels) and reported according to STROBE guidance [13].

### 2.6 Ethics

Ethics approval for use of de-identified linked administrative data was granted by the Monash University Human Research Ethics Committee (approval number 11681) and relevant data custodians; the requirement for individual consent was waived.

## 3 Results

### 3.1 Cohort and incidence

Of 16,415 eligible first ambulance-attended AF events, 43 (0.3%) lacked remoteness classification and were excluded from stratified analyses, leaving 16,372 events: 11,914 (72.8%) in major cities, 3691 (22.5%) in inner regional areas, and 767 (4.7%) in outer regional/remote areas. Transport to hospital occurred in 11,840 (99.4%), 3667 (99.3%), and 765 (99.7%) events, respectively (*P* = 0.44). Linked public ED records were available for 10,209 (85.7%), 3140 (85.1%), and 636 (82.9%) events, respectively (*P* = 0.086).

**Fig. 1:**
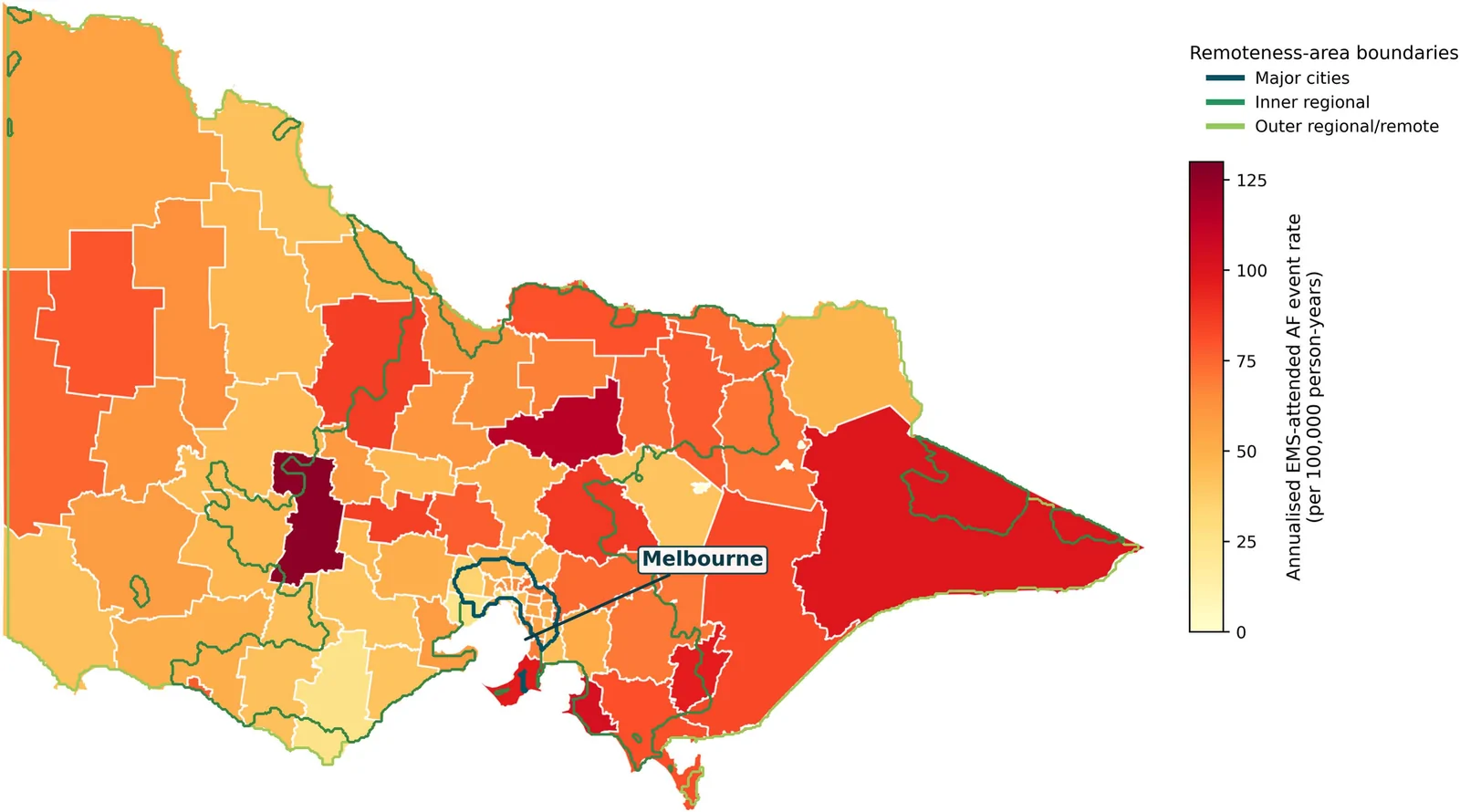
Annualised EMS-attended AF event rate and remoteness context in Victoria. Victorian local government areas (LGAs) are shaded by annualised EMS-attended AF event rate based on patient LGA of residence. Coloured boundary lines on the same map show the ASGS Edition 3 Remoteness Areas collapsed to the study categories of major cities, inner regional, and outer regional/remote. Remoteness boundaries do not necessarily coincide with LGA boundaries. EMS = emergency medical services. AF = atrial fibrillation. LGA = local government area.

Incidence differed by remoteness (Fig. 1). Major cities had 54.2 events per 100,000 person-years (95% CI 53.2–55.2), compared with 71.3 (69.0–73.7) in inner regional areas and 68.1 (63.3–73.0) in outer regional/remote areas. Compared with major cities, IRRs were 1.31 (95% CI 1.27–1.36; *P* < 0.001) for inner regional areas and 1.25 (95% CI 1.16–1.35; *P* < 0.001) for outer regional/remote areas. The LGA-level map showed substantial geographic variation within broad remoteness categories, underlining that incidence was not uniform across non-metropolitan Victoria.

### 3.2 Baseline characteristics

Median age was similar but slightly lower with increasing remoteness, and the proportion of female patients decreased from 58.2% in major cities to 53.5% in inner regional areas and 51.9% in outer regional/remote areas (*P* < 0.001; Table 1). Socioeconomic disadvantage increased with remoteness: 19.9% of major city events were in the most disadvantaged IRSD quintile compared with 28.9% in inner regional and 39.6% in outer regional/remote areas (*P* < 0.001).

**Table 1:** Selected baseline characteristics by remoteness of residence.

Characteristic	Major cities (*n* = 11,914)	Inner regional (*n* = 3691)	Outer regional/remote (*n* = 767)	*P* value
Age, years, median [IQR]	77 [67–84]	76 [67–83]	75 [68–82]	0.001
Female, n (%)	6934 (58.2)	1976 (53.5)	398 (51.9)	<0.001
Most disadvantaged IRSD quintile, n (%)	2156 (19.9)	1031 (28.9)	281 (39.6)	<0.001
Pre-existing AF, n (%)	4873 (40.9)	1571 (42.6)	292 (38.1)	0.042
Hypertension, n (%)	6232 (52.3)	1845 (50.0)	375 (48.9)	0.014
Heart failure, n (%)	1424 (12.0)	343 (9.3)	69 (9.0)	<0.001
Diabetes, n (%)	2184 (18.3)	560 (15.2)	112 (14.6)	<0.001
COPD, n (%)	911 (7.6)	354 (9.6)	65 (8.5)	<0.001
High thromboembolic risk (CHA2DS2-VASc ≥ 2 in men or ≥3 in women), n (%)	9386 (78.8)	2879 (78.0)	603 (78.6)	0.39
Initial heart rate ≥100 beats/min, n (%)	8654 (72.8)	2653 (72.1)	537 (70.4)	0.020
Initial systolic BP <90 mmHg, n (%)	600 (5.0)	155 (4.2)	37 (4.8)	0.29
Initial GCS <15, n (%)	1488 (12.5)	286 (7.8)	51 (6.7)	<0.001

AF = atrial fibrillation. BP = blood pressure. COPD = chronic obstructive pulmonary disease. GCS = Glasgow Coma Scale. IRSD=Index of Relative Socioeconomic Disadvantage. Percentages use available denominators; full baseline characteristics are provided in the Supplementary Appendix.

Clinical risk profiles were broadly comparable. Approximately four-fifths of patients in each group had high thromboembolic risk by CHA2DS2-VASc category (*P* = 0.39), and more than 70% had an initial heart rate of at least 100 beats/min. Comorbidity differences were generally small in absolute terms, although heart failure and diabetes were recorded more often in major cities, whereas COPD was slightly more frequent in inner regional areas. Hypotension at initial assessment was uncommon and did not differ materially across remoteness categories (*P* = 0.29).

### 3.3 Prehospital and hospital pathways

Prehospital time intervals differed across geography (Table 2). Response time and scene duration were longer outside major cities (both *P* < 0.001), and total prehospital time from call initiation to ED arrival increased from a median of 1.0 h in major cities to 1.2 h in both regional categories (*P* < 0.001). Median transport duration was 20 min in all groups (*P* = 0.452), but this summary measure obscured a long-tail distribution in regional Victoria.

**Table 2:** Selected prehospital and hospital pathway measures by remoteness of residence.

Measure	Major cities	Inner regional	Outer regional/remote	P value
Transported to hospital, n (%)	11,840 (99.4)	3667 (99.3)	765 (99.7)	0.44
Response time, min, median [IQR]	12.1 [8.9–18.3]	13.8 [9.2–21.9]	14.7 [8.8–26.2]	<0.001
Scene duration, min, median [IQR]	18.0 [13.0–24.0]	21.0 [15.0–28.0]	22.0 [16.4–30.0]	<0.001
Transport duration, min, median [IQR]	20.0 [14.0–28.0]	20.0 [10.0–39.0]	20.0 [8.0–40.0]	0.45
Call to ED arrival, h, median [IQR]	1.0 [0.9–1.2]	1.2 [0.9–1.5]	1.2 [0.8–1.7]	<0.001
Transport duration >60 min, n (%)	81 (0.7)	232 (6.4)	84 (11.2)	<0.001
Ambulance time at ED, min, median [IQR]	47.0 [37.0–59.0]	38.0 [28.0–51.0]	34.0 [25.0–46.9]	<0.001
Offload delay ≥ 40 min, n (%)	7950 (69.6)	1576 (46.2)	249 (35.9)	<0.001
ED length of stay, h, median [IQR]	4.4 [3.2–7.6]	4.8 [3.3–7.7]	4.5 [3.2–7.4]	0.011
Hospital admission, n (%)	8192 (80.2)	2206 (70.3)	465 (73.1)	<0.001
ED discharge without hospital admission, n (%)	2017 (19.8)	934 (29.7)	171 (26.9)	<0.001

ED = emergency department. IQR = interquartile range. Hospital admission includes admitted care captured in hospital data, including short-stay unit care where captured. ED discharge without hospital admission is calculated among transported events with linked ED disposition data.

Transport duration exceeding 60 min occurred in 0.7% of major city events, 6.4% of inner regional events, and 11.2% of outer regional/remote events (*P* < 0.001). After adjustment, the odds of transport duration exceeding 60 min were markedly higher for inner regional (aOR 10.55, 95% CI 8.01–13.90; *P* < 0.001) and outer regional/remote areas (aOR 21.79, 95% CI 15.47–30.69; *P* < 0.001) than for major cities (Table 3). Thus, although most transported patients had short-to-moderate transport times, prolonged transport was a distinct regional and remote care-pathway feature.

**Table 3:** Adjusted associations between remoteness of residence and selected outcomes.

Outcome	Inner regional vs major cities, aOR (95% CI)	P value	Outer regional/remote vs major cities, aOR (95% CI)	P value
Transport duration >60 min	10.55 (8.01–13.90)	<0.001	21.79 (15.47–30.69)	<0.001
Offload delay ≥ 40 min	0.38 (0.35–0.41)	<0.001	0.23 (0.20–0.28)	<0.001
Hospital admission	0.57 (0.52–0.63)	<0.001	0.64 (0.52–0.77)	<0.001
Direct-current cardioversion	1.09 (0.95–1.24)	0.226	0.96 (0.72–1.28)	0.789
Catheter ablation	1.00 (0.84–1.18)	0.957	1.00 (0.70–1.43)	0.995
EMS re-attendance within 30 days	0.99 (0.80–1.24)	0.950	0.80 (0.49–1.28)	0.347
EMS re-attendance within 6 months	0.94 (0.78–1.13)	0.494	0.65 (0.42–1.01)	0.055
Death within 30 days	0.89 (0.73–1.07)	0.211	1.00 (0.70–1.42)	0.982
Death within 12 months	0.91 (0.81–1.02)	0.112	1.07 (0.86–1.34)	0.556

aOR = adjusted odds ratio. Models adjusted for age, sex, pre-existing AF, coronary artery disease, chronic obstructive pulmonary disease, hypertension, heart failure, diabetes, cerebrovascular disease, peripheral vascular disease, area-level IRSD quintile, and calendar year, using complete cases. Major cities are the reference group. Model sample sizes are reported in the Supplementary Appendix.

**Fig. 2:**
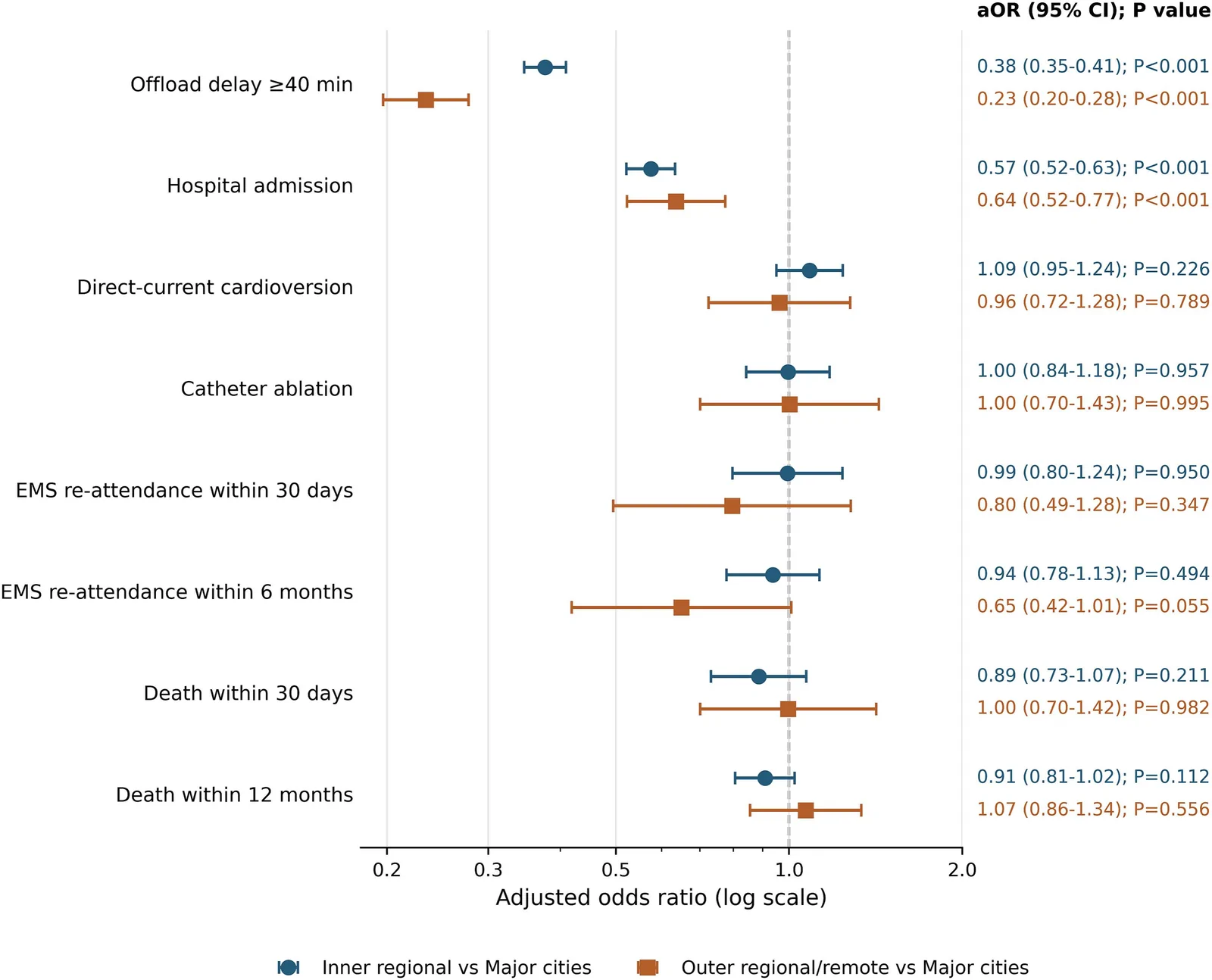
Adjusted odds ratios for selected care-pathway and clinical outcomes by remoteness of residence. Adjusted odds ratios compare inner regional and outer regional/remote areas with major cities. Models use the covariates listed in Table 3. Transport duration greater than 60 min is not displayed because its large effect estimates compress the remainder of the plot; estimates are reported in Table 3.

By contrast, ambulance offload delay was concentrated in major cities. Offload delay occurred in 69.6% of major city events, compared with 46.2% in inner regional and 35.9% in outer regional/remote events (*P* < 0.001). Relative to major cities, offload delay remained less likely in inner regional (aOR 0.38, 95% CI 0.35–0.41; *P* < 0.001) and outer regional/remote areas (aOR 0.23, 95% CI 0.20–0.28; *P* < 0.001; Fig. 2). ED length of stay differed modestly, with medians of 4.4 h, 4.8 h, and 4.5 h across the three groups (*P* = 0.011).

Hospital admission was more frequent in major cities (80.2%) than inner regional (70.3%) or outer regional/remote areas (73.1%; *P* < 0.001). Compared with major cities, adjusted odds of admission were lower in inner regional (aOR 0.57, 95% CI 0.52–0.63; *P* < 0.001) and outer regional/remote areas (aOR 0.64, 95% CI 0.52–0.77; *P* < 0.001). Conversely, ED discharge without hospital admission occurred in 19.8%, 29.7%, and 26.9% of transported events, respectively (*P* < 0.001). Because transport was almost universal, these differences translated into a sizeable group of regional patients who travelled to hospital and returned home without inpatient admission.

Emergency department disposition differed in the same direction. Among transported events with a linked ED departure status, 17.9% overall were discharged directly home from the ED and 34.9% were managed in an ED short-stay unit. Direct discharge home was more common outside major cities (15.3%, 24.9%, and 24.4%; *P* < 0.001), whereas short-stay unit care was concentrated in major cities (38.5%, 27.5%, and 14.2%; *P* < 0.001; Supplementary Table S3).

### 3.4 Rhythm-control procedures and outcomes

Over available follow-up, direct-current cardioversion was recorded in 9.7% of major city events, 10.2% of inner regional events, and 8.5% of outer regional/remote events (*P* = 0.35); catheter ablation was recorded in 6.5%, 6.2%, and 5.2%, respectively (*P* = 0.30). In adjusted models, neither cardioversion nor catheter ablation differed by remoteness: for inner regional areas, the aORs were 1.09 (95% CI 0.95–1.24; *P* = 0.226) and 1.00 (0.84–1.18; *P* = 0.957), respectively; for outer regional/remote areas, they were 0.96 (0.72–1.28; *P* = 0.789) and 1.00 (0.70–1.43; *P* = 0.995), respectively (Table 3). Restricting procedures to a fixed 12-month window gave cardioversion rates of 7.4%, 7.7%, and 6.4% and catheter ablation rates of 3.7%, 3.2%, and 2.9%, with no association with remoteness after adjustment (cardioversion aOR 1.08, 95% CI 0.93–1.26 and 0.99, 0.72–1.36; catheter ablation 0.88, 0.71–1.10 and 0.92, 0.57–1.48).

EMS re-attendance for AF within 30 days occurred in 3.1%, 3.2%, and 2.6% of events in major cities, inner regional areas, and outer regional/remote areas, respectively (*P* = 0.69); re-attendance within 6 months occurred in 4.9%, 4.7%, and 3.4% (*P* = 0.15). Relative to major cities, adjusted odds of 30-day re-attendance were 0.99 (95% CI 0.80–1.24; *P* = 0.950) in inner regional and 0.80 (0.49–1.28; *P* = 0.347) in outer regional/remote areas. Corresponding 6-month estimates were 0.94 (0.78–1.13; *P* = 0.494) and 0.65 (0.42–1.01; *P* = 0.055).

Mortality at 30 days was 5.0%, 4.6%, and 5.6% across the three groups (*P* = 0.39), and mortality at 12 months was 15.2%, 14.1%, and 15.8% (*P* = 0.18). Relative to major cities, adjusted odds of 30-day mortality were 0.89 (95% CI 0.73–1.07; *P* = 0.211) in inner regional and 1.00 (0.70–1.42; *P* = 0.982) in outer regional/remote areas. Corresponding 12-month estimates were 0.91 (0.81–1.02; *P* = 0.112) and 1.07 (0.86–1.34; *P* = 0.556). The absence of measurable outcome differences should be interpreted alongside the substantial differences in transport burden and ED disposition.

Inferences for hospital admission and for both rhythm-control procedures were unchanged when standard errors were clustered by receiving ED campus (Supplementary Table S11).

### 3.5 Socioeconomic disadvantage

Socioeconomic disadvantage was associated with care and outcomes independently of remoteness (Supplementary Table S10). Compared with the least disadvantaged IRSD quintile, patients in the most disadvantaged quintile were less likely to undergo catheter ablation (aOR 0.46, 95% CI 0.36–0.58; *P* < 0.001) or direct-current cardioversion (0.67, 0.56–0.81; *P* < 0.001), and were more likely to be re-attended by EMS within 30 days (1.44, 1.06–1.95; *P* = 0.018) and to die within 30 days (1.70, 1.31–2.21; *P* < 0.001) and 12 months (1.46, 1.24–1.72; *P* < 0.001). Prolonged transport, offload delay, and hospital admission did not differ by IRSD quintile (all *P* > 0.10). Adjustment for IRSD moved the regional estimates for rhythm-control procedures towards the null, most visibly for catheter ablation, where the aOR for outer regional/remote areas changed from 0.81 in models without IRSD to 1.00 in the final models (Supplementary Table S12).

## 4 Discussion

This statewide linked cohort study identified four findings relevant to rural and regional acute cardiovascular care. First, population-based incidence of ambulance-attended AF was about 25–30% higher in regional areas than in major cities. Second, travel burden increased sharply with remoteness, particularly for transport lasting more than 60 min. Third, acute-care bottlenecks differed by geography, with metropolitan patients experiencing more offload delay whereas regional patients experienced longer transport and more ED discharge after transport. Fourth, measured clinical outcomes and rhythm-control procedure rates were similar after adjustment, despite these pathway differences [4], [5].

The higher incidence of ambulance-attended AF in regional areas likely reflects interacting factors rather than a discrete biological threshold. Regional populations often have different age structures, cardiovascular risk profiles, socioeconomic conditions, and access to primary and ambulatory care. AF management depends on longitudinal stroke-risk assessment, anticoagulation, symptom management, and follow-up; when these services are less accessible, symptomatic AF may be more likely to trigger emergency help-seeking rather than community-based assessment [1], [2], [3], [6], [7]. The incidence findings therefore should not be read simply as evidence of more arrhythmia, but as evidence of greater EMS-attended AF burden.

Socioeconomic disadvantage increased progressively with remoteness, with 39.6% of outer regional/remote events arising from the most disadvantaged IRSD quintile, and disadvantage was associated with care and outcomes independently of geography. Patients in the most disadvantaged quintile had less than half the odds of catheter ablation and around 50% higher odds of death at 30 days and 12 months than those in the least disadvantaged quintile. Because IRSD is an area-level measure, these associations cannot establish an individual causal pathway; they do, however, identify an overlap between geographic and socioeconomic disadvantage that a purely geographic framing would miss. Economic vulnerability may amplify the burden of long-distance care through travel costs, time away from work or caregiving, and reduced capacity to access follow-up. Recent AF research identified social-vulnerability clusters in which economic disadvantage was associated with adverse cardiovascular outcomes, and a contemporary Canadian study reported socioeconomic and geographic inequities in access to AF ablation despite publicly funded hospital care [14], [15]. Notably, the modest regional shortfall in catheter ablation in our cohort attenuated to the null once IRSD was included, which suggests that socioeconomic composition rather than distance alone accounts for much of it. Although adjusted procedure rates were similar by remoteness, the linked data do not capture referral attempts, waiting times, travel, or decisions not to pursue specialist care because of cost or distance.

A journey of more than an hour was almost unheard of in major cities and commonplace in outer regional and remote Victoria, affecting fewer than one in a hundred events against more than one in ten. The median transport time of 20 min was identical in all three groups and hid this entirely, because the burden sits in the tail of the distribution rather than at its centre. For patients, prolonged transport can mean disruption, out-of-pocket costs, time away from family or carers, and difficulty returning home after discharge. For EMS systems, long transports also remove ambulance capacity from the originating community and can increase reliance on neighbouring branches or volunteer resources. These opportunity costs are not captured by mortality or re-attendance outcomes but are central to rural service planning.

Comparable rhythm-control procedure rates are encouraging but should be interpreted cautiously. Similar rates may indicate that hospital pathways are broadly accessible across remoteness categories, especially given comparable haemodynamics, stroke-risk profiles, and mortality. However, administrative data do not show where procedures were performed, waiting times, travel distance, referral attempts, private-sector care, or patient barriers to specialist assessment. A patient in regional Victoria may ultimately receive cardioversion or ablation at a similar measured rate but experience substantially greater time, cost, and travel burden. Equivalent procedure rates therefore do not prove equivalent access or care experience, and future analyses should incorporate facility-level data and patient-reported measures.

Differences between receiving hospitals are a further plausible contributor to the admission and procedure patterns we observed. Patients in this cohort presented to 35 public ED campuses and were subsequently admitted to 85 hospitals, which differ in size, teaching status, and on-site access to cardiology and electrophysiology services. Facility-level capability data were not available to us, and the receiving hospital is itself partly determined by remoteness, so adjusting for hospital would remove part of the exposure of interest. We therefore treated destination hospital as a source of correlated error and repeated the admission and rhythm-control models with standard errors clustered by receiving ED campus, which did not change any inference. Linking service-capability data, such as the presence of a dedicated heart rhythm unit or an on-site electrophysiology laboratory, would allow the question to be addressed directly and would also show where procedures are performed and how far patients travel to reach them.

The inverse pattern of metropolitan offload delay highlights that statewide AF pathways must be adaptable. Interventions targeting access block and ED congestion in major cities may not solve rural transport burden, while rural risk-stratification pathways may not address metropolitan offload. Telemedicine may be especially useful as enabling infrastructure in rural AF pathways, linking paramedics or local clinicians with cardiology or virtual ED support for ECG review, treatment decisions, anticoagulation planning, and rapid follow-up [16], [17], [18], [19]. The MIRACLE-AF trial supports the potential of village-doctor-led, telemedicine-supported integrated AF care in older rural patients, although it was not an acute EMS diversion study [19]. Any Victorian prehospital model would therefore require prospective safety evaluation, defined escalation thresholds, anticoagulation governance, equitable digital access, and reliable local follow-up. In metropolitan areas, reducing ED crowding and improving transfer of care may have greater marginal value for EMS availability, whereas in regional settings the highest-yield intervention may be better selection of patients who truly require transport and better follow-up for those safely managed outside hospital.

The policy relevance of AF extends beyond this single condition. AF-related hospitalisations have increased in Australia, and cardiovascular disease remains a major contributor to national health burden [20], [21]. Acute AF presentations therefore sit at the intersection of chronic disease management, emergency access, and rural health service design. A pathway that safely avoids unnecessary transport for selected low-risk AF patients could release EMS capacity, reduce patient travel burden, and allow ED resources to be focused on patients with high-risk features or decompensated comorbid disease.

The finding that 26.9–29.7% of regional transports ended in ED discharge without hospital admission is particularly important. Some ED discharges are clinically appropriate and necessary, especially where AF symptoms require ECG confirmation, rate control, assessment of anticoagulation, or exclusion of myocardial ischaemia, infection, heart failure, or other precipitants. Nevertheless, for a remote patient, a long ambulance journey followed by discharge from ED may represent avoidable burden if safe assessment, treatment, and follow-up can be provided closer to home. This issue is not merely about reducing hospital activity; it is about avoiding low-value travel while preserving safety for patients who need urgent hospital care.

Structured prehospital risk stratification could therefore have particular value in regional settings. One example is the STAY (Safe Treatment of Atrial fibrillation in the communitY) model, in which paramedics identify low-risk AF presentations using a structured assessment, initiate treatment when indicated with cardiologist support, and arrange rapid outpatient review so eligible patients can remain at home [22]. Such pathways would be most relevant for haemodynamically stable patients without high-risk features, and should not replace transport for patients with syncope, ongoing chest pain, severe dyspnoea, hypotension, suspected sepsis, acute heart failure, or other red flags. Implementation would require robust governance, reliable follow-up, anticoagulation support, access to ECG review, integration with primary care, and clear escalation options.

The observation of similar EMS re-attendance and mortality across remoteness categories is reassuring, but it should not lead to the conclusion that geographic equity has been achieved. Mortality and EMS re-attendance are relatively blunt endpoints for a condition in which the major burdens include symptoms, anxiety, treatment uncertainty, travel, waiting, and disrupted follow-up. They also do not capture unplanned primary care visits, privately funded care, medication delays, or the experience of carers. A rural patient who travels for an hour to hospital, waits, receives rate control, and is discharged home may have the same 30-day survival as a metropolitan patient, but a very different care burden.

### 4.1 Study limitations

This study has limitations. Routinely collected administrative data may be affected by coding variability and do not capture symptom burden, AF subtype, anticoagulation prescribing, frailty, reasons for transport or admission, or patient preferences. Because cohort ascertainment relied on paramedic clinical documentation, some AF episodes may have been misclassified; however, ECG-confirmed AF was required when the primary paramedic diagnosis was “arrhythmia”, and misclassification is more likely to arise from paroxysmal AF that had terminated before paramedic arrival, which would under-count events. Remoteness was based on residential address, not necessarily incident location, and collapsing five ASGS categories into three groups may obscure heterogeneity within regional Victoria. Socioeconomic position was measured at area level and does not describe individual circumstances. Follow-up ended on a fixed date, so its length varied by index date and 12.4% of events had less than 12 months of potential follow-up for procedures; the fixed 12-month sensitivity analysis gave consistent results. ED length-of-stay and discharge analyses relied on linked public ED data and therefore represent pathway measures among records with available ED linkage rather than complete capture of every destination. Although the final models adjusted for individual measured comorbidities and area-level socioeconomic disadvantage, residual confounding remains possible.

Destination-hospital characteristics, including academic status, cardiology or electrophysiology capability, dedicated rhythm services, and local admission practice, were not available for adjustment. These facility-level factors could partly explain differences in admission or procedure rates, and future multilevel analyses should jointly model patient remoteness and hospital capability. The linked data also did not capture all primary care contacts, private outpatient care, referral attempts, waiting time, or travel burden for specialist interventions.

## 5 Conclusions

Regional and remote residence in Victoria was associated with higher incidence of ambulance-attended AF and longer prehospital intervals, whereas major city residence was associated with greater offload delay. Short-term outcomes and rhythm-control procedure rates were similar across remoteness categories after adjustment, but these findings should be interpreted alongside the practical burden of prolonged transport and ED discharge after long-distance travel, and alongside a socioeconomic gradient in rhythm-control procedures and mortality that cut across geography. Statewide AF care pathways should therefore be locally adapted. Metropolitan strategies should prioritise ED flow and offload delay, while rural and regional models should prioritise safe prehospital risk stratification, transport avoidance for carefully selected low-risk patients, timely follow-up, and reliable anticoagulation and cardiology support.

## Data sharing

The linked administrative data used for this study are not publicly available because they contain potentially re-identifiable health information and are governed by Victorian data custodian approvals. Access may be considered by the relevant data custodians subject to ethics approval and data-sharing agreements.

## CRediT authorship contribution statement

**Malanka Lankaputhra:** Writing – review & editing, Writing – original draft, Formal analysis. **Emily Mahony:** Data curation. **Luke P. Dawson:** Writing – review & editing, Methodology, Formal analysis. **Aleksandr Voskoboinik:** Writing – review & editing. **Riley Batchelor:** Writing – review & editing. **Vishal Goel:** Writing – review & editing. **Mark Horrigan:** Writing – review & editing, Conceptualization. **Peter Kistler:** Writing – review & editing, Data curation, Conceptualization. **Jonathan Kalman:** Writing – review & editing, Data curation, Conceptualization. **Tegwyn McManamny:** Writing – review & editing. **David Anderson:** Writing – review & editing, Data curation. **Emily Nehme:** Writing – review & editing. **Ziad Nehme:** Writing – review & editing, Supervision, Conceptualization. **Dion Stub:** Writing – review & editing, Supervision, Methodology, Conceptualization. **Jocasta Ball:** Writing – review & editing, Supervision, Conceptualization.

## Funding

This work was supported by Ambulance Victoria. ML and RB are supported by National Health and Medical Research Council postgraduate scholarships. ZN and DS are supported by NHMRC Investigator Grants. DS is also supported by a National Heart Foundation Future Leader Fellowship. The funders had no role in study design, data analysis, interpretation, or manuscript preparation.

## Declaration of competing interest

The authors declare that they have no known competing financial interests or personal relationships that could have appeared to influence the work reported in this paper.
