# Supplementary material for: Remoteness and emergency care pathways for ambulance-attended atrial fibrillation: a population-based linked cohort study
Source: Int J Cardiol Heart Vasc. 2026 Aug 20;66:101997. doi: 10.1016/j.ijcha.2026.101997 (PMC13524516; doi:10.1016/j.ijcha.2026.101997)
Supplement: Supplementary file 1 [file mmc1.docx]

**Supplementary Appendix**

**Remoteness and emergency care pathways for ambulance-attended atrial fibrillation: a population-based linked cohort study**

This appendix contains cohort derivation, detailed descriptive tables, revised adjusted estimates, secondary and sensitivity analyses, model sample sizes, and a STROBE checklist. In response to peer review, all multivariable models were rerun without CHA2DS2-VASc category or Charlson Comorbidity Index, and two new analyses were added (Tables S10 and S11).

**Figure S1. Cohort derivation**

| **Stage** | **n** |
| --- | --- |
| Eligible first ambulance-attended AF events in the linked dataset | 16,415 |
| Excluded: missing remoteness of residence | 43 (0.3%) |
| Cohort with remoteness classification | 16,372 |
| Major cities | 11,914 (72.8%) |
| Inner regional | 3,691 (22.5%) |
| Outer regional/remote | 767 (4.7%) |

AF=atrial fibrillation.

**Table S1. Full baseline characteristics by remoteness of residence**

| **Characteristic** | **Major cities** | **Inner regional** | **Outer regional/remote** | **P value** |
| --- | --- | --- | --- | --- |
| n | 11,914 | 3,691 | 767 |  |
| Age, years, median [IQR] | 77 [67-84] | 76 [67-83] | 75 [68-82] | 0.001 |
| Female, n (%) | 6934 (58.2) | 1976 (53.5) | 398 (51.9) | <0.001 |
| ***Index of Relative Socioeconomic Disadvantage, n (%)*** |  |  |  |  |
| 1 (most disadvantaged) | 2156 (19.9) | 1031 (28.9) | 281 (39.6) | <0.001* |
| 2 | 1874 (17.3) | 1026 (28.7) | 239 (33.7) |  |
| 3 | 2202 (20.3) | 795 (22.3) | 132 (18.6) |  |
| 4 | 2396 (22.1) | 482 (13.5) | 47 (6.6) |  |
| 5 (least disadvantaged) | 2212 (20.4) | 237 (6.6) | 11 (1.5) |  |
| ***Pre-existing medical conditions, n (%)*** |  |  |  |  |
| Atrial fibrillation | 4873 (40.9) | 1571 (42.6) | 292 (38.1) | 0.042 |
| Coronary artery disease† | 1925 (16.2) | 641 (17.4) | 109 (14.2) | 0.058 |
| Coronary artery bypass grafting | 1316 (11.0) | 416 (11.3) | 77 (10.0) | 0.61 |
| Chronic obstructive pulmonary disease | 911 (7.6) | 354 (9.6) | 65 (8.5) | <0.001 |
| Hypertension | 6232 (52.3) | 1845 (50.0) | 375 (48.9) | 0.014 |
| Heart failure | 1424 (12.0) | 343 (9.3) | 69 (9.0) | <0.001 |
| Diabetes | 2184 (18.3) | 560 (15.2) | 112 (14.6) | <0.001 |
| Cerebrovascular disease | 1092 (9.2) | 288 (7.8) | 57 (7.4) | 0.015 |
| Peripheral vascular disease | 140 (1.2) | 44 (1.2) | 6 (0.8) | 0.60 |
| Renal disease‡ | 652 (5.5) | 144 (3.9) | 35 (4.6) | 0.001 |
| ***CHA2DS2-VASc category, n (%)*** |  |  |  |  |
| Low risk (0 in men, 1 in women) | 884 (7.4) | 258 (7.0) | 53 (6.9) | 0.39* |
| Moderate risk (1 in men, 2 in women) | 1644 (13.8) | 554 (15.0) | 111 (14.5) |  |
| High thromboembolic risk (>=2 in men, >=3 in women) | 9386 (78.8) | 2879 (78.0) | 603 (78.6) |  |
| ***Symptoms, n (%)*** |  |  |  |  |
| Palpitations | 4056 (34.0) | 1167 (31.6) | 214 (27.9) | <0.001 |
| Chest pain | 3198 (26.8) | 1102 (29.9) | 180 (23.5) | <0.001 |
| Dizziness | 2750 (23.1) | 800 (21.7) | 146 (19.0) | 0.011 |
| Shortness of breath | 4236 (35.6) | 1432 (38.8) | 285 (37.2) | 0.001 |
| ***Vital signs*** |  |  |  |  |
| Initial heart rate, beats/min, mean (SD) | 119.93 (34.06) | 119.80 (33.99) | 117.45 (33.66) | 0.078 |
| Initial heart rate <50 beats/min, n (%) | 296 (2.5) | 69 (1.9) | 12 (1.6) | 0.020* |
| Initial heart rate 50-99 beats/min, n (%) | 2939 (24.7) | 958 (26.0) | 214 (28.0) |  |
| Initial heart rate >=100 beats/min, n (%) | 8654 (72.8) | 2653 (72.1) | 537 (70.4) |  |
| Initial systolic BP, mm Hg, mean (SD) | 132.45 (28.02) | 133.51 (27.67) | 131.45 (26.49) | 0.053 |
| Initial systolic BP <90 mm Hg, n (%) | 600 (5.0) | 155 (4.2) | 37 (4.8) | 0.29* |
| Initial systolic BP 90-119 mm Hg, n (%) | 2978 (25.1) | 879 (23.9) | 192 (25.1) |  |
| Initial systolic BP 120-139 mm Hg, n (%) | 3218 (27.1) | 1027 (27.9) | 209 (27.4) |  |
| Initial systolic BP 140-159 mm Hg, n (%) | 2870 (24.1) | 921 (25.0) | 198 (25.9) |  |
| Initial systolic BP >=160 mm Hg, n (%) | 2221 (18.7) | 700 (19.0) | 128 (16.8) |  |
| Respiratory rate <12 breaths/min, n (%) | 17 (0.1) | 4 (0.1) | 1 (0.1) | 0.47* |
| Respiratory rate 12-20 breaths/min, n (%) | 9346 (78.6) | 2945 (79.9) | 599 (78.3) |  |
| Respiratory rate >=21 breaths/min, n (%) | 2535 (21.3) | 735 (20.0) | 165 (21.6) |  |
| GCS, mean (SD) | 14.78 (0.88) | 14.88 (0.63) | 14.87 (0.71) | <0.001 |
| Temperature, degrees C, mean (SD) | 36.63 (0.82) | 36.65 (0.83) | 36.61 (0.83) | 0.57 |
| ***Prehospital treatment, n (%)*** |  |  |  |  |
| Any medical treatment from paramedics | 6939 (58.2) | 2430 (65.8) | 501 (65.3) | <0.001 |
| Aspirin | 2383 (20.0) | 786 (21.3) | 145 (18.9) | 0.15 |
| Analgesics | 1794 (15.1) | 544 (14.7) | 99 (12.9) | 0.26 |
| Amiodarone | 47 (0.4) | 15 (0.4) | 2 (0.3) | 0.84 |
| Adenosine | 19 (0.2) | 11 (0.3) | 1 (0.1) | 0.22 |
| Synchronised cardioversion by paramedics§ | 11 (0.1) | 6 (0.2) | 0 (0.0) | 0.34 |

BP=blood pressure. GCS=Glasgow Coma Scale. IQR=interquartile range. SD=standard deviation. Percentages use available denominators. *P value for the distribution across all categories of the variable. †Coronary artery disease combines recorded coronary artery disease and ischaemic heart disease; adjusted models used the coronary artery disease field. ‡Chronic kidney disease was documented in fewer than 0.1% of prehospital records, which reflects prehospital documentation rather than true prevalence, so renal disease identified from admitted-care diagnostic coding is reported instead. §Cardioversion delivered by paramedics before hospital arrival; cardioversion at any point during the index episode or subsequent admitted care is reported in Table S5. The main manuscript reports a condensed baseline table and presents GCS as GCS <15 to improve clinical interpretability.

**Table S2. Incidence of EMS-attended AF by remoteness of residence**

| **Remoteness** | **Cases (2015 to Jun 2019)** | **Incidence per 100,000 person-years (95% CI)** | **IRR vs major cities (95% CI)** |
| --- | --- | --- | --- |
| Major cities | 11,914 | 54.2 (53.2-55.2) | 1.00 (reference) |
| Inner regional | 3,691 | 71.3 (69.0-73.7) | 1.31 (1.27-1.36) |
| Outer regional/remote | 767 | 68.1 (63.3-73.0) | 1.25 (1.16-1.35) |

AF=atrial fibrillation. CI=confidence interval. EMS=emergency medical services. IRR=incidence rate ratio.

**Table S3. Prehospital and hospital pathway measures by remoteness of residence**

| **Measure** | **Major cities** | **Inner regional** | **Outer regional/remote** | **P value** |
| --- | --- | --- | --- | --- |
| Transported to hospital, n (%) | 11840 (99.4) | 3667 (99.3) | 765 (99.7) | 0.435 |
| Response time, min, median [IQR] | 12.1 [8.9-18.3] | 13.8 [9.2-21.9] | 14.7 [8.8-26.2] | <0.001 |
| Scene duration, min, median [IQR] | 18.0 [13.0-24.0] | 21.0 [15.0-28.0] | 22.0 [16.4-30.0] | <0.001 |
| Transport duration, min, median [IQR] | 20.0 [14.0-28.0] | 20.0 [10.0-39.0] | 20.0 [8.0-40.0] | 0.452 |
| Call start to ED arrival, h, median [IQR] | 1.0 [0.9-1.2] | 1.2 [0.9-1.5] | 1.2 [0.8-1.7] | <0.001 |
| Transport duration >60 min, n (%) | 81 (0.7) | 232 (6.4) | 84 (11.2) | <0.001 |
| Ambulance time at ED, min, median [IQR] | 47.0 [37.0-59.0] | 38.0 [28.0-51.0] | 34.0 [25.0-46.9] | <0.001 |
| Offload delay >=40 min, n (%) | 7950 (69.6) | 1576 (46.2) | 249 (35.9) | <0.001 |
| ED length of stay, h, median [IQR] | 4.4 [3.2-7.6] | 4.8 [3.3-7.7] | 4.5 [3.2-7.4] | 0.011 |
| Hospital admission, n (%) | 8192 (80.2) | 2206 (70.3) | 465 (73.1) | <0.001 |
| Discharged directly home from ED, n (%) | 1566 (15.3) | 782 (24.9) | 155 (24.4) | <0.001 |
| Managed in an ED short-stay unit, n (%) | 3939 (38.5) | 864 (27.5) | 90 (14.2) | <0.001 |

ED=emergency department. IQR=interquartile range. Hospital admission includes admitted care captured in hospital data, including short-stay unit care where captured. ED disposition categories are calculated among transported events with a linked ED departure status (n=10,229, 3,143, and 636, respectively).

**Table S4. Adjusted comparisons in prehospital and hospital outcomes by remoteness of residence**

| **Outcome** | **Comparison** | **Adjusted OR (95% CI)** | **P value** |
| --- | --- | --- | --- |
| Transport duration >60 min | Inner regional vs major cities | 10.55 (8.01-13.90) | <0.001 |
| Transport duration >60 min | Outer regional/remote vs major cities | 21.79 (15.47-30.69) | <0.001 |
| Offload delay >=40 min | Inner regional vs major cities | 0.38 (0.35-0.41) | <0.001 |
| Offload delay >=40 min | Outer regional/remote vs major cities | 0.23 (0.20-0.28) | <0.001 |
| Hospital admission | Inner regional vs major cities | 0.57 (0.52-0.63) | <0.001 |
| Hospital admission | Outer regional/remote vs major cities | 0.64 (0.52-0.77) | <0.001 |

Models adjusted for age, sex, pre-existing AF, coronary artery disease, chronic obstructive pulmonary disease, hypertension, heart failure, diabetes, cerebrovascular disease, peripheral vascular disease, area-level IRSD quintile, and calendar year, using complete cases. Model n=14,865 for transport duration >60 min, n=14,232 for offload delay >=40 min, and n=12,744 for hospital admission.

**Table S5. Unadjusted rhythm-control procedures by remoteness of residence**

| **Outcome** | **Major cities, n (%)** | **Inner regional, n (%)** | **Outer regional/remote, n (%)** | **P value** |
| --- | --- | --- | --- | --- |
| ***Over available follow-up*** |  |  |  |  |
| Direct-current cardioversion | 1160 (9.7) | 375 (10.2) | 65 (8.5) | 0.35 |
| Catheter ablation | 780 (6.5) | 230 (6.2) | 40 (5.2) | 0.30 |
| ***Within a fixed 12-month window*** |  |  |  |  |
| Direct-current cardioversion | 879 (7.4) | 283 (7.7) | 49 (6.4) | 0.44 |
| Catheter ablation | 444 (3.7) | 118 (3.2) | 22 (2.9) | 0.20 |

Procedures were identified from admitted-care procedure codes. Follow-up ended on a fixed date, so its length varied by index date (median 2.7 years, IQR 1.6 to 3.9 years), and 12.4% of events had less than 12 months of potential follow-up.

**Table S6. Adjusted associations between remoteness of residence and rhythm-control procedures**

| **Outcome** | **Comparison** | **Adjusted OR (95% CI)** | **P value** |
| --- | --- | --- | --- |
| ***Over available follow-up*** |  |  |  |
| Direct-current cardioversion | Inner regional vs major cities | 1.09 (0.95-1.24) | 0.226 |
| Direct-current cardioversion | Outer regional/remote vs major cities | 0.96 (0.72-1.28) | 0.789 |
| Catheter ablation | Inner regional vs major cities | 1.00 (0.84-1.18) | 0.957 |
| Catheter ablation | Outer regional/remote vs major cities | 1.00 (0.70-1.43) | 0.995 |
| ***Within a fixed 12-month window (sensitivity analysis)*** |  |  |  |
| Direct-current cardioversion | Inner regional vs major cities | 1.08 (0.93-1.26) | 0.308 |
| Direct-current cardioversion | Outer regional/remote vs major cities | 0.99 (0.72-1.36) | 0.964 |
| Catheter ablation | Inner regional vs major cities | 0.88 (0.71-1.10) | 0.260 |
| Catheter ablation | Outer regional/remote vs major cities | 0.92 (0.57-1.48) | 0.721 |

Models adjusted for the same covariates as Table S4 and used complete cases (n=14,987).

**Table S7. Unadjusted clinical outcomes by remoteness of residence**

| **Outcome** | **Major cities, n (%)** | **Inner regional, n (%)** | **Outer regional/remote, n (%)** | **P value** |
| --- | --- | --- | --- | --- |
| EMS re-attendance within 30 days | 368 (3.1) | 118 (3.2) | 20 (2.6) | 0.69 |
| EMS re-attendance within 6 months | 585 (4.9) | 173 (4.7) | 26 (3.4) | 0.15 |
| Death within 30 days | 591 (5.0) | 168 (4.6) | 43 (5.6) | 0.39 |
| Death within 12 months | 1814 (15.2) | 519 (14.1) | 121 (15.8) | 0.18 |

EMS=emergency medical services. Death within 30 days is defined as death within 30 days inclusive of the index event date, matching the outcome used in the adjusted models.

**Table S8. Adjusted associations between remoteness of residence and clinical outcomes**

| **Outcome** | **Comparison** | **aOR (95% CI)** | **P value** |
| --- | --- | --- | --- |
| EMS re-attendance within 30 days | Inner regional vs major cities | 0.99 (0.80-1.24) | 0.950 |
| EMS re-attendance within 30 days | Outer regional/remote vs major cities | 0.80 (0.49-1.28) | 0.347 |
| EMS re-attendance within 6 months | Inner regional vs major cities | 0.94 (0.78-1.13) | 0.494 |
| EMS re-attendance within 6 months | Outer regional/remote vs major cities | 0.65 (0.42-1.01) | 0.055 |
| Death within 30 days | Inner regional vs major cities | 0.89 (0.73-1.07) | 0.211 |
| Death within 30 days | Outer regional/remote vs major cities | 1.00 (0.70-1.42) | 0.982 |
| Death within 12 months | Inner regional vs major cities | 0.91 (0.81-1.02) | 0.112 |
| Death within 12 months | Outer regional/remote vs major cities | 1.07 (0.86-1.34) | 0.556 |

aOR=adjusted odds ratio. Models adjusted for the same covariates as Table S4 and used complete cases (n=14,987).

**Table S9. STROBE checklist for cohort studies**

| **Item** | **Recommendation** | **Reported in manuscript** |
| --- | --- | --- |
| 1 Title and abstract | Indicate study design and provide an informative summary | Title; Abstract |
| 2 Background/rationale | Explain scientific background and rationale | Introduction |
| 3 Objectives | State specific objectives | Introduction |
| 4 Study design | Present key elements of study design | Methods: Study design and data sources |
| 5 Setting | Describe setting, locations, dates, and follow-up | Methods: Study design and data sources; Outcomes |
| 6 Participants | Describe eligibility criteria and selection | Methods; Results; Figure S1 |
| 7 Variables | Define outcomes, exposures, predictors, and confounders | Methods: Remoteness classification; Socioeconomic status; Outcomes; Statistical analysis |
| 8 Data sources/measurement | Describe data sources and measurement methods | Methods: Study design and data sources; Outcomes |
| 9 Bias | Describe efforts to address potential bias | Methods; Discussion: Study limitations |
| 10 Study size | Explain study size | Results; Figure S1 |
| 11 Quantitative variables | Explain handling of quantitative variables | Methods: Statistical analysis |
| 12 Statistical methods | Describe statistical methods and handling of confounding | Methods: Statistical analysis |
| 13 Participants | Report numbers at each stage | Results; Figure S1 |
| 14 Descriptive data | Describe participant characteristics and missing data | Results; Tables 1 and S1 |
| 15 Outcome data | Report outcome events | Results; Tables 2-3 and S3-S8 |
| 16 Main results | Give unadjusted and adjusted estimates with precision | Results; Table 3; Tables S4-S8 |
| 17 Other analyses | Report subgroup and sensitivity analyses if done | Results; Tables S5, S6, S10, S11, and S12 |
| 18 Key results | Summarise key results with reference to objectives | Discussion |
| 19 Limitations | Discuss limitations | Discussion: Study limitations |
| 20 Interpretation | Provide cautious interpretation | Discussion |
| 21 Generalisability | Discuss external validity | Discussion: Study limitations |
| 22 Funding | Give source and role of funders | Funding |

**Table S10. Association between area-level socioeconomic disadvantage and selected outcomes**

| **Outcome** | **aOR (95% CI)** | **P value** |
| --- | --- | --- |
| Transport duration >60 min | 0.82 (0.54-1.23) | 0.34 |
| Offload delay >=40 min | 1.02 (0.91-1.15) | 0.69 |
| Hospital admission | 1.11 (0.96-1.29) | 0.146 |
| Direct-current cardioversion | 0.67 (0.56-0.81) | <0.001 |
| Catheter ablation | 0.46 (0.36-0.58) | <0.001 |
| EMS re-attendance within 30 days | 1.44 (1.06-1.95) | 0.018 |
| EMS re-attendance within 6 months | 0.94 (0.74-1.21) | 0.649 |
| Death within 30 days | 1.70 (1.31-2.21) | <0.001 |
| Death within 12 months | 1.46 (1.24-1.72) | <0.001 |

aOR=adjusted odds ratio comparing the most disadvantaged with the least disadvantaged Index of Relative Socioeconomic Disadvantage quintile. Models adjusted for age, sex, remoteness category, pre-existing AF, coronary artery disease, chronic obstructive pulmonary disease, hypertension, heart failure, diabetes, cerebrovascular disease, peripheral vascular disease, and calendar year, using complete cases. Quintile 5 (least disadvantaged) is the reference group.

**Table S11. Sensitivity analysis with standard errors clustered by receiving emergency department campus**

| **Outcome** | **Inner regional vs major cities, aOR (95% CI)** | **Outer regional/remote vs major cities, aOR (95% CI)** |
| --- | --- | --- |
| Hospital admission | 0.57 (0.44-0.75) | 0.64 (0.49-0.82) |
| Direct-current cardioversion | 1.06 (0.82-1.37) | 1.04 (0.81-1.34) |
| Catheter ablation | 1.10 (0.88-1.37) | 1.24 (0.92-1.69) |

aOR=adjusted odds ratio. Models use the covariates listed in Table S4, restricted to events with a linked emergency department campus (n=12,744 for hospital admission and n=12,764 for rhythm-control procedures), with standard errors clustered by receiving campus. Patients presented to 35 public emergency department campuses and were subsequently admitted to 85 hospitals. Major cities are the reference group.

**Table S12. Remoteness estimates from models with and without adjustment for socioeconomic disadvantage**

| **Outcome** | **Inner regional, aOR without IRSD** | **Inner regional, aOR with IRSD** | **Outer regional/remote, aOR without IRSD** | **Outer regional/remote, aOR with IRSD** |
| --- | --- | --- | --- | --- |
| Transport duration >60 min | 10.03 | 10.55 | 20.07 | 21.79 |
| Offload delay >=40 min | 0.38 | 0.38 | 0.24 | 0.23 |
| Hospital admission | 0.59 | 0.57 | 0.66 | 0.64 |
| Direct-current cardioversion | 1.03 | 1.09 | 0.87 | 0.96 |
| Catheter ablation | 0.89 | 1.00 | 0.81 | 1.00 |
| EMS re-attendance within 30 days | 1.05 | 0.99 | 0.89 | 0.80 |
| EMS re-attendance within 6 months | 0.94 | 0.94 | 0.65 | 0.65 |
| Death within 30 days | 0.97 | 0.89 | 1.15 | 1.00 |
| Death within 12 months | 0.97 | 0.91 | 1.18 | 1.07 |

aOR=adjusted odds ratio. IRSD=Index of Relative Socioeconomic Disadvantage. Both model sets adjust for age, sex, calendar year, and the individual comorbidities listed in Table S4; the second set additionally adjusts for IRSD quintile. Major cities are the reference group. Confidence intervals for the models with IRSD are given in Tables S4, S6, and S8.
